# Supplementary material for: Engineering of NEMO as calcium indicators with large dynamics and high sensitivity
Source: Nat Methods. 2023 Apr 20;20(6):918–24. doi: 10.1038/s41592-023-01852-9 (PMC10250196; doi:10.1038/s41592-023-01852-9)
Supplement: Supplementary file 1 — Supplementary Tables 1–6. [file 41592_2023_1852_MOESM1_ESM.pdf]

nature methods

Article

https://doi.org/10.1038/s41592-023-01852-9

Engineering of NEMO as calcium indicators with large dynamics and high sensitivity

In the format provided by the authors and unedited

Supplementary Table 1. *In cellulo* screening results of constructs with a GCaMP-like design$^{\$}$

No	Name*	N terminal mutations	CBP**	L1***	cp-FP mutations	Mutations in CaM	F_0 (afu)	Dynamic range	N #	Primer ##
1	**GCaMP6m**	/	M13	LE	Template	Template	609±44	24.7±1.2	137/48	
2	**Kozak-GCaMP6m**	Template	M13	LE	/	/	927±24	22.9±0.3	1058/304	66,67
3	**jGCaMP8f**	6×his-TR	NOSP-N10T-S15I-S17R	LKI	K18H	Q41L-G76M-S77K	692±29	7.5±0.1	298/100	
4	**jGCaMP8s**	6×his-TR	NOSP-N10T-S15I-S17M	LKI	K18H	F12Y-Q41H-G76M-S77K	872±41	6.1±0.1	362/128	
5	**jGCaMP8m**	6×his-TR	NOSP-N10T-S15I-A16G-S17R	LKI	K18H	F12Y-G76M-S77K	764±34	7.6±0.1	352/105	
6	**NEMO-0.1**	/	M13	LE	mNG(146-236)-GGTGGS-mNG(1-145)	/	5405±435	1.2±0.01	25/25	1~4,62~65
7	**NEMO-0.2**	/	M13	LE	mNG(148-236)-GGTGGS-mNG(1-147)	/	3143±697	1.1±0.02	17/17	1~8
8	**NEMO-0.3**	/	M13	LE	mNG(160-236)-GGTGGS-mNG(1-159)	/	0	0	30/30	1~4,9~12

Note:

$^{\$}$, diagram showing the design of GCaMP-like GECIs

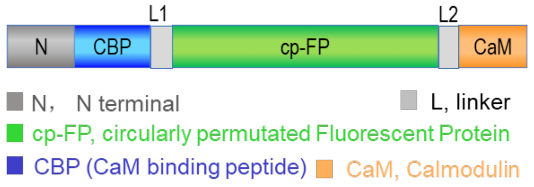

/, NA

*, Kozak: Kozak consensus sequence was used

**, NOSP: Nitric Oxide Synthase Peptide CaM binding domain for jGCaMP8 variants.

***, L1, Linker between CBP and cp-FP

#, N=A/B: A, number of cells used for measurements of the basal fluorescence，B, number of cells used for calculating the dynamic ranges.

##, Primer, See Supplementary Table 6 for primer sequences corresponding to numbering.

Supplementary Table 2. *In cellulo* screening results of NEMO constructs with a NCaMP7-like design$^{\$}$

No	Name+ (All contain Kozak)	NES-L1*	Mutations in CaM	L3**	CBP***#	#L4	##FP mutations	F_0 (afu)	Dynamic range	N ###	Primer ####
1	**NCaMP7**	/	Template	GGSGGGSSS	M13	MYF	Template	3499±194	15.8±0.5	205/86	
2	**NCaMP7-with 6×his tag**	/	/	GGSGGGSSS	M13	MYF	/	4925±372	17.9±0.4	149/56	68,24
3	**Neo-linker-0**	/	/	/	M13	MYF	/	717±51	56.1±4.1	47/20	13,14,15,24
4	**Neo-linker-1**	/	/	G	M13	MYF	/	365±72	150.0±4.9	38/35	13,14,16,24
5	**Neo-linker-2**	/	/	GG	M13	MYF	/	348±53	205.9±4.2	45/38	13,14,17,24
6	**Neo-linker-3**	/	/	GGS	M13	MYF	/	187±13	157.8±8.8	116/14	13,14,18,24
7	**Neo-linker-4**	/	/	GGGS	M13	MYF	/	586±58	80.6±9.4	82/20	13,14,19,24
8	**Neo-linker-5**	/	/	GGGGS	M13	MYF	/	526±35	154.5±4.8	175/59	13,14,20,24
9	**Neo-linker-6**	/	/	GGSGGS	M13	MYF	/	952±43	95.1±1.6	538/173	13,14,21,24
10	**Neo-linker-7**	/	/	GGGSGGS	M13	IYF	/	280±14	237.2±3.0	246/108	13,14,38,24
11	**Neo-linker-8**	/	/	GGGSGGGS	M13	MYF	/	625±53	48.1±4.1	68/43	13,14,23,24
12	**5-Q3D**	/	Q3D	GGGGS	M13	MYF	/	675±98	107.9±8.1	34/34	13,25,33,24
13	**5-A15E**	/	A15E	GGGGS	M13	MYF	/	272±46	134.1±9.8	32/32	13,26,34,24
14	**5-A15L**	/	A15L	GGGGS	M13	MYF	/	603±69	61.6±2.1	72/72	13,27,35,24
15	**5-A15D**	/	A15D	GGGGS	M13	MYF	/	546±71	44.9±1.7	44/44	13,28,36,24
16	**5-A15Q**	/	A15Q	GGGGS	M13	MYF	/	469±83	127.6±11.2	40/40	13,29,37,24

No	Name+ (All contain Kozak)	NES-L1*	Mutations in CaM	L3**	CBP***#	#L4	##FP mutations	F_0 (afu)	Dynamic range	N ###	Primer ####
**17**	**5-M36F**	/	M36F	GGGGS	M13	MYF	/	566±46	94.0±7.2	50/50	13,30,38,24
**18**	**5-Q41L**	/	Q41L	GGGGS	M13	MYF	/	533±40	202.1±8.1	186/186	13,31,39,24
**19**	**5-Q3D-A15E**	/	Q3D-A15E	GGGGS	M13	MYF	/	143±17	106.3±4.7	30/30	13,26,34,24
**20**	**5-Q3D-M36F**	/	Q3D-M36F	GGGGS	M13	MYF	/	725±65	76.1±1.9	75/75	13,30,38,24
**21**	**5-E14A-A15E**	/	E14A-A15E	GGGGS	M13	MYF	/	224±29	70.5±6.9	22/22	13,41,42,24
**22**	**5-A15E-M36F**	/	A15E-M36F	GGGGS	M13	MYF	/	312±48	91.1±5.5	34/34	13,30,38,24
**23**	**5-A15L-M36F**	/	A15L-M36F	GGGGS	M13	MYF	/	2102±168	69.5±1.4	185/67	13,30,38,24
**24**	**5-Q3D-A15L**	/	Q3D-A15L	GGGGS	M13	MYF	/	2023±191	59.8±2.1	194/77	13,27,35,24
**25**	**5f**	/	Q3D-A15E-M36F	GGGGS	M13	MYF	/	521±35	79.4±0.9	219/112	13,30,38,24
**26**	**5-Q3D-A15L-M36F**	/	Q3D-A15L-M36F	GGGGS	M13	MYF	/	1827±109	43.8±0.5	211/80	13,30,38,24
**27**	**5f-Q41L**	/	Q3D-A15E-M36F-Q41L	GGGGS	M13	MYF	/	445±24	96.7±1.8	187/58	51,52,53,24
**28**	**5f-N42S**	/	Q3D-A15E-M36F-N42S	GGGGS	M13	MYF	/	256±31	72.3±2.4	27/27	13,44,46,24
**29**	**5-8f-2**	/	/	GGGGS	NOSP-N10T-S15I-S17R	MYF	/	1849±220	12.6±0.35	56/56	13,47,48,24
**30**	**5-jGCaMP8f**	/	Q41L	GGGGS	NOSP-N10T-S15I-S17R	MYF	/	1448±161	15.0±0.5	48/48	13,31,39,24
**31**	**5f-ckkap-LHWL**	/	/	GGGGS	ckkap-LHWL	MYF	/	638±164	1.1±0.01	14/14	13,49,50,24
**32**	**6f**	/	Q3D-A15E-M36F	GGSGGS	M13	MYF	/	430±52	81.8±4.5	33/33	13,14,21,24
**33**	**6f-Q41L**	/	Q3D-A15E-M36F-Q41L	GGSGGS	M13	MYF	/	490±42	81.1±1.2	175/52	13,31,39,24
**34**	**7f**	/	Q3D-A15E-M36F	GGGSGGS	M13	IYF	/	231±22	172.9±6.9	63/63	13,30,38,24
**35**	**7f-Q41L**	/	Q3D-A15E-M36F-Q41L	GGGSGGS	M13	IYF	/	419±38	164.7±3.1	162/46	13,31,39,24
**36**	**7f-N42S**	/	Q3D-A15E-M36F-N42S	GGGSGGS	M13	IYF	/	229±31	155.4±4.5	35/35	13,44,46,24
**37**	**7-I324M**	/	/	GGGSGGS	M13	MYF	/	455±32	137.1±1.8	57/57	13,14,22,24
**38**	**NCaMP7-MI**	/	/	GGSGGGSSS	M13	IYF	/	1198±56	170.8±3.2	785/235	13,32,40,24
**39**	**NCaMP7-MI-A15E**	/	A15E	GGSGGGSSS	M13	IYF	/	246±16	172.7±4.6	142/52	13,26,34,24
**40**	**NCaMP7-MI-A15D**	/	A15D	GGSGGGSSS	M13	IYF	/	437±32	122.8±2.4	168/48	13,28,36,24
**41**	**NCaMP7-MI-E14A-A15E**	/	E14A-A15E-	GGSGGGSSS	M13	IYF	/	129±9	176.8±3.3	159/54	13,41,42,24
**42**	**NCaMP7-MI-Q3D-A15E**	/	Q3D-A15E	GGSGGGSSS	M13	IYF	/	165±13	148.7±2.4	148/45	13,25,33,24
**43**	**NCaMP7-MI-A15E-M36F**	/	A15E-M36F	GGSGGGSSS	M13	IYF	/	184±11	192.4±6.1	174/52	13,30,38,24
**44**	**NCaMP7-MI-Q3D-A15E-M36F**	/	Q3D-A15E-M36F	GGSGGGSSS	M13	IYF	/	302±22	147.2±2.5	156/56	13,30,38,24
**45**	**5-MI (NEMOc)**	/	/	GGGGS	M13	IYF	/	445±25	422.2±15.3	195/57	13,32,40,24
**46**	**5-A15L-Q3D-MI**	/	Q3D-A15L	GGGGS	M13	IYF	/	603±39	236.9±6.4	257/65	13,32,40,24
**47**	**5f-MI**	/	Q3D-A15E-M36F	GGGGS	M13	IYF	/	178±11	266.2±8.2	144/52	13,32,40,24
**48**	**5-A15L-Q3D-M36F-MI**	/	Q3D-A15L-M36F	GGGGS	M13	IYF	/	557±32	279.8±7.4	268/65	13,32,40,24
**49**	**6-MI**	/	/	GGSGGS	M13	IYF	/	322±21	280.1±5.7	148/56	13,32,40,24

No	Name[+] (All contain Kozak)	NES-L1[*]	Mutations in CaM	L3[**]	CBP[***#]	[#]L4	[##]FP mutations	F_0 (afu)	Dynamic range	N [###]	Primer [####]
**50**	**NCaMP7-MI-ΔC10**	/	/	GGSGGGSSS	M13	IYF	**ΔC10**	1720±103	32.9±0.7	218/64	13,54
**51**	**5-MI-ΔC10**	/	/	GGGGS	M13	IYF	**ΔC10**	362±35	214.6±7.9	121/34	13,54
**52**	**NES-Neo6**	1	/	GGSGGS	M13	MYF	/	1078±88	49.2±1.7	83/29	51,52,53,24
**53**	**NES-Neo7**	1	/	GGGSGGS	M13	IYF	/	681±83	189.8±8.2	27/27	51,52,53,24
**54**	**NES-Neo5f**	1	Q3D-A15E-M36F	GGGGS	M13	MYF	/	639±40	73.1±1.3	166/60	51,52,53,24
**55**	**NES-Neo7f**	1	Q3D-A15E-M36F	GGGSGGS	M13	IYF	/	268±28	150.1±4.7	17/17	51,52,53,24
**56**	**NES-6-MI (NEMOm)**	1		GGSGGS	M13	IYF	/	1085±93	240.7±7.6	260/90	51,52,53,24
**57**	**NES-7f-Q41L**	1	Q3D-A15E-M36F-Q41L	GGGSGGS	M13	IYF	/	531±32	144.7±4.5	255/83	51,52,53,24
**58**	**NES-5f-Q41L**	1	Q3D-A15E-M36F-Q41L	GGGGS	M13	MYF	/	593±38	76.6±2.8	245/67	51,52,53,24
**59**	**NES-5-Q41L**	1	Q41L	GGGGS	M13	MYF	/	936±54	124.1±4.9	301/104	51,52,53,24
**60**	**NES-5f-MI (NEMOf)**	1	Q3D-A15E-M36F	GGGGS	M13	IYF	/	508±51	245.5±8.6	200/59	51,52,53,24
**61**	**NES-NCaMP7-MI (NEMOb)**	1	/	GGSGGGSSS	M13	IYF	/	2230±95	128.8±3.1	629/139	51,52,53,24
**62**	**NES-5-MI**	1	/	GGGGS	M13	IYF	/	220±20	384.1±16.7	158/49	51,52,53,24
**63**	**NES-5-A15E-MI**	1	A15E	GGGGS	M13	IYF	/	114±7	423.5±17.3	134/43	53,26,34,24
**64**	**NES-5-E14A-A15E-MI**	1	E14A-A15E	GGGGS	M13	IYF	/	143±13	304.8±11.8	166/50	53,41,42,24
**65**	**NES-NeoCaMP5-A15L-Q3D**	1	Q3D-A15L	GGGGS	M13	MYF	/	1562±117	36.6±0.9	169/64	51,52,53,24
**66**	**NES-NeoCaMP5-A15L-Q3D-M36F**	1	Q3D-A15L-M36F	GGGGS	M13	MYF	/	2025±96	39.4±1.2	199/55	51,52,53,24
**67**	**NES-NCaMP7-MI-ΔC10**	1	/	GGSGGGSSS	M13	IYF	**ΔC10**	4141±233	28.8±1.2	198/56	53,54
**68**	**NES-5-A15E-MI-ΔC10**	1	A15E	GGGGS	M13	IYF	**ΔC10**	152±12	212.3±6.2	91/43	53,54
**69**	**NES-5f-MI-ΔC10**	1	Q3D-A15E-M36F	GGGGS	M13	IYF	**ΔC10**	235±15	72.2±3.8	106/34	53,54
**70**	**NES-6-MI-ΔC10**	1	/	GGSGGS	M13	IYF	**ΔC10**	1807±178	90.6±3.8	183/55	13,54
**71**	**NES2-NCaMP7-MI (NEMOs)**	2	/	GGSGGGSSS	M13	IYF	/	2010±151	102.3±4.0	294/83	52,53,55,56,24
**72**	**NES2-NCaMP7-MI-ΔC10**	2	/	GGSGGGSSS	M13	IYF	**ΔC10**	2440±210	15.5±0.6	154/47	53,54

Note:

[$], diagram showing the design of NCaMP7-like GECIs

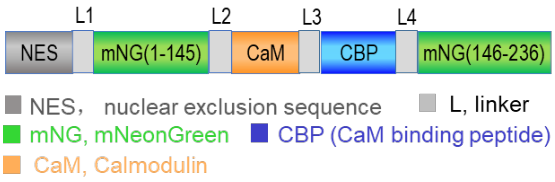

/: NA

[+], Kozak: Kozak consensus sequence was used

*, NES-L1 sequence:

1: MLQNELALKLAGLDINKTG-GGS

2: MLQNELALKLAGLDINKTG-GITLGMDELYK

[**], L3, Linker between CaM and CBP

[***], Nitric Oxide Synthase Peptide CaM binding domain (NOSP)-N10T-S15I-S17R

[#], L4, Linker between CBP and cpEGFP or mNG

##, ΔC10, 10 amino acids were truncated from the C-terminus of mNG-146-236

###, N=A/B: A, number of cells used for measurements of the basal fluorescence, B, number of cells used for calculating the dynamic ranges

####, Primer numbers, See Supplementary Table 6 for primer sequences with corresponding numbers.

Supplementary Table 3. *In vitro* Ca^{2+} titration results and kinetics of NEMO variants

Sensor	Mg^{2+}	K_d (nM)	Hill number	F_{min} (10 mM EGTA)	F_{rest} (100 nM Ca^{2+})	F_{max} (38 μM Ca^{2+})	Dynamic range (F_{max}/F_{min})	K_{off} (s^{-1})	K_{on}[1] ($*10^6$, $M^{-1}s^{-1}$)
GCaMP6m	-	174.3±6.1	2.5±0.2	155±6	700±49	4023±93	26.7±0.9	2.48±0.02	14.20
	+	206.4±5	2±0.1	176±4	680±34	4133±85	23.6±0.6	2.52±0.02	12.21
GCaMP6f	-	354.4±1.5	2±0.2	228±4	515.8±8.9	8019±252	35.2±1.3	3.72±0.004	10.49
	+	568.8±9.4	1.7±0.0	268±3	477.8±4.4	8250±212	30.7±0.6	4.35±0.03	7.65
NCaMP7	-	97.9±2	2.5±0.1	406±29	16870±1964	32753±2394	80±3.2	3.29±0.01	33.56
	+	98.3±1.5	2.4±0.1	677±45	16216±1325	30628±1927	45.5±0.9	2.48±0.01	25.23
NEMOc	-	492.8±15.9	3.4±0.3	98±4	152±4	15875±236	165.3±7.7	4.59±0.03	9.31
	+	557.3±15.5	3.8±0.4	89±7	137±6	15267±578	182.2±12.6	3.16±0.02	5.67
NEMOm	-	198.9±4.8	3.3±0.2	123±5	1735±150	19396±725	159.2±4.3	2.22±0.01	11.17
	+	248.4±3.7	3.3±0.1	134±6	1065±21	19706±520	149.1±3.5	1.46±0.01	5.88
NEMOf	-	440.1±19.4	2.9±0.3	118±1	205±13	16705±411	141.5±3.8	6.73±0.04	15.30
	+	528.4±17	3.2±0.3	118±2	304±53	15085±265	127.9±2.3	5.57±0.04	10.54
NEMOs	-	129.1±3.1	3.1±0.2	65±3	2572±254	8351±194	131.3±3.4	2.26±0.01	17.48
	+	155.8±3.8	2.7±0.2	87±5	1890±115	8489±311	99.7±2.9	1.46±0.01	9.37
NEMOb	-	198.7±2.6	2.5±0.1	340±1	3880±89	27829±713	81.9±2.1	1.96±0.003	9.85
	+	141±1.3	2.6±0.1	414±4	8019±54	27857±408	67.4±1.3	1.56±0.01	11.06

Note: [1] K_{on} was calculated from K_{off} and K_d with this equation: $K_{on} = K_{off}/K_d$

Supplementary Table 4. *In vitro* biophysical properties of NEMO sensors

Sensor	[*1]Mg^{2+}	Ca^{2+}	ρ[*2]		ε_{max} ($mM^{-1}*cm^{-1}$)		Φ		F_1[*3] ($mM^{-1}*cm^{-1}$)		pKa
			Anionic	Neutral	Anionic	Neutral	Anionic	Neutral	Anionic	Neutral	
		+	0.47±0.00	0.53±0.00	82.18±1.48	40.60±0.65	0.65	0.14	25.10±0.45	3.01±0.05	7.18±0.09
GCaMP6m	-	-	0.05±0.05	0.95±0.01	60.89±12.06	40.10±1.35	0.38	0.09	1.15±0.05	3.42±0.13	/
		+	0.46±0.02	0.54±0.02	77.99±2.95	38.16±4.43	0.66	0.14	23.66±1.22	2.87±0.26	7.27±0.17
	+	-	0.04±0.01	0.96±0.01	85.96±13.50	35.89±1.56	0.38	0.09	1.11±0.13	3.11±0.11	/
		+	0.84±0.02	0.16±0.02	116.40±2.20	42.22±4.71	0.75	0.16	73.67±2.70	1.03±0.05	6.07±0.62
NCaMP7	-	-	0.21±0.01	0.79±0.01	14.99±0.33	51.92±2.50	0.35	0.08	1.12±0.07	3.26±0.10	/
		+	0.85±0.02	0.15±0.02	107.10±0.91	38.60±3.12	0.72	0.18	65.63±1.73	1.02±0.04	4.93±0.33
	+	-	0.22±0.01	0.78±0.01	15.29±1.26	52.16±1.95	0.40	0.08	1.34±0.06	3.26±0.16	/
		+	0.85±0.02	0.15±0.02	113.20±3.44	40.31±1.14	0.67	0.18	64.54±3.02	1.08±0.08	5.07±0.33
NEMOc	-	-	0.20±0.02	0.80±0.02	7.35±1.13	49.56±0.91	0.29	0.06	0.43±0.06	2.37±0.01	/
		+	0.86±0.01	0.14±0.01	111.40±0.91	41.81±1.67	0.67	0.17	64.26±2.67	0.99±0.05	4.97±0.43
	+	-	0.19±0.01	0.81±0.01	7.68±0.25	50.07±1.59	0.15	0.07	0.22±0.01	2.84±0.06	/

Note: [*1] The effect of Mg^{2+} was tested because the free cytosolic Mg^{2+} concentration is in the range of mM and the presence of Mg^{2+} might affect the dynamic range of NCaMP7 (Table 1 in Subach et al, 2020), the parental template of NEMO sensors. [*2] ρ is the relative concentration of chromophore; [*3] F_1 is one-photon brightness defined as the product of ρ, ε and Φ.

Supplementary Table 5. Two-photon biophysical properties of NEMO sensors *in vitro*.

Sensor	Mg^{2+}	Ca^{2+}	σ_2[*1] (GM)		F_2[*2] (GM)	
			Anionic	Neutral	Anionic	Neutral
GCaMP6m	+	+	18.18±1.2	24.41±1.55	5.52±0.37	1.85±0.12
NCaMP7	+	+	42.37±4.13	16.9±3.69	25.93±2.53	0.46±0.10
NEMOc	+	+	51.94±6.07	21.59±5.01	29.93±3.50	0.51±0.12

Note: [*1] σ_2 is two-photon cross-sections. [*2]F_2 is two-photon brightness defined as the product of ρ, Φ and σ_2.; ρ, Φ are from Supplementary table 4.

Supplementary Table 6. Sequences of primers used to generate GECI constructs listed in Supplementary Table 1&2.

No.	Sequence (5' to 3')
1	ACCGAGCTCGGATCCGCCACCATGGGTTCTCATCATCATC
2	GCTCCCTCCGGTACCGCCCTTGTACAGCTCGTCCATGC
3	GGCGGTACCGGAGGGAGCATGGTGTCCAAGGGCGAAGA
4	TGGATATCTGCAGAATTCTCACTTCGCTGTCATCAT
5	TTCTTGCTTCTGCACCACTCGAGTGAGCTCAGCCGACCTATAG
6	CTATAGGTCGGCTGAGCTCACTCGAGTGGTGCAGAAGCAAGAA
7	TCAGTCAGTTGGTCCGGCAGGTCGGCGGCTGTCAGGCT
8	GACAGCCGCCGACCTGCCGGACCAACTGACTGAAGAGC
9	AAGGTGCTGATGATGGTCTCGAGTGAGCTCAGCCGACCTATAG
10	CTATAGGTCGGCTGAGCTCACTCGAGACCATCATCAGCACCTT
11	GCTCTTCAGTCAGTTGGTCCGGCAGCTTGTCATTGGGGTAGGT
12	CCCCAATGACAAGCTGCCGGACCAACTGACTGAAGAGC
13	GGTACCGAGCTCGGATCCGCCACCATGCATCATCATCATCATCATGTGAGCAAGGGCGA
14	CTTGGCGGTCATCATCTG
15	CAGATGATGACCGCCAAGAGAAGAAAATGGAAT
16	CAGATGATGACCGCCAAGGGCAGAAGAAAATGGAAT
17	CAGATGATGACCGCCAAGGGCGGCAGAAGAAAATGGAAT
18	CAGATGATGACCGCCAAGGGCGGCAGCAGAAGAAAATGGAAT
19	CAGATGATGACCGCCAAGGGCGGCGGAAGCAGAAGAAAATGGAAT
20	CAGATGATGACCGCCAAGGGCGGCGGAGGCAGCAGAAGAAAATGGAAT
21	CAGATGATGACCGCCAAGGGCGGCAGCGGAGGAAGCAGAAGAAAATGGAAT
22	CAGATGATGACCGCCAAGGGCGGCGGCAGCGGAGGAAGCAGAAGAAAATGGAAT
23	CAGATGATGACCGCCAAGGGCGGCGGCAGCGGAGGAGGAAGCAGAAGAAAATGGAAT
24	CTGGATATCTGCAGAATTCTTACTTGTACAGCTCGTCCAT
25	TCTTCTGTCAGATCGTCGTGGGCTT
26	AGGCTAAACTCCTCCTTGAA
27	AGGCTAAACAGCTCCTTGAAT
28	TGTCGAACAGGCTAAAGTCCTCCTTGAATTCGGC
29	TGTCGAACAGGCTAAATTGCTCCTTGAATTCGGC
30	GGCCCAGGCTTCTGAACACTGTGCCCAGC
31	TCTGTGGGATTAAGGCCCAGGCTTCT
32	TCGGCGAAGTAGATGCTAGAAAGTC
33	AAGCCCACGACGATCTGACAGAAGA
34	TTCAAGGAGGAGTTTAGCCTG
35	TTCAAGGAGCTGTTTAGCCT
36	GCCGAATTCAAGGAGGACTTTAGCCTGTTCGACA
37	GCCGAATTCAAGGAGCAATTTAGCCTGTTCGACA
38	TGGGCACAGTGTTCAGAAGCCTGGGCCA
39	AGAAGCCTGGGCCTTAATCCCACAGA
40	TTTCTAGCATCTACTTCGCCGA
41	ACAGGCTAAACTCGGCCTTGAATTCGGC
42	AATTCAAGGCCGAGTTTAGCCTGTTCGACA
43	TCTGTGGGATTAAGGCCCAGGCTTCT
44	TCTGTGGGACTTTGGCCCAGG
45	AGAAGCCTGGGCCTTAATCCCACAGA
46	CCTGGGCCAAAGTCCCACAGA
47	GGCGGTGGCCACCTCCTTGAAGGTCTTCTTGCGGCTGCCTCCGCCGCCCTTGGCGGTCA
48	GGAGGTGGCCACCGCCGTGAAGATCATCGCCCGCCTGATGGGCATGTACTTCGCCGACT
49	TGACCAGGATCACAGTGGTCAGGCTGGGGATGTGCTTGACGCTGCCTCCGCCGCCCTTG
50	CTGTGATCCTGGTCAAGTCTATGCTGAGAAAGCGTTCCTTTGGAAACCCATTTATGTACTTCGCCGACTG
51	GGACTTGATATTAACAAGACTGGAGGAGGTTCTCATCATCATCATCATCATGTGAGCAA
52	ATGCTGCAGAACGAGCTTGCTCTTAAGTTGGCTGGACTTGATATTAACAAG

**53**	GGTACCGAGCTCGGATCCGCCACCATGCTGCAGAACGAGC
**54**	CTGTGCTGGATATCTGCAGAATTCTTAGGTGAAGGCCTTCTGCCACTC
**55**	TGGACGAGCTTTACAAGATGCATCATCATCATCATCATGTGAGCAAGGGC
**56**	CTTGATATTAACAAGACTGGAGGGATCACTCTCGGCATGGACGAGCTTTACAAG
**57**	GTGGTGGTGGTGGTGGTGCTCGAGTTACTTGTACAGCTCGTCCATGCCCAT
**58**	TGGAGGAGAACCCAGGCCCAATGCATCATCATCATCATCATGTGAGCAAGGGCGA
**59**	ATGGTGTCTAAGGGCGAAGAGCTGA
**60**	TGGGCCTGGGTTCTCCTCCACGTCGCCGGC
**61**	CTTTAAGAAGGAGATATACCATGCATCATCATCATCATCATGTGAGCAAGGGCGA
**62**	TTCTTGCTTCTGCACCAGTCGGCCTCGAGTGAGCTCAGCCGACCTATAG
**63**	CTATAGGTCGGCTGAGCTCACTCGAGGCCGACTGGTGCAGAAGCAAGA
**64**	TCAGTCAGTTGGTCCGGCAGGGCTGTCAGGCTATTGGT
**65**	ACCAATAGCCTGACAGCCCTGCCGGACCAACTGACTGA
**66**	GCTGGATATCTGCAGAATTCTTACTTCGCTGTCATCAT
**67**	ACCGAGCTCGGATCCGCCACCATGGGTTCTCATCATCATC
**68**	GAGTTCGTGACCGCCGCCGAGGAGGAGAACATGG
