# Supplementary material for: Engineering of NEMO as calcium indicators with large dynamics and high sensitivity
Source: Nat Methods. 2023 Apr 20;20(6):918–24. doi: 10.1038/s41592-023-01852-9 (PMC10250196; doi:10.1038/s41592-023-01852-9)
Supplement: Supplementary file 2 — Reporting Summary [file 41592_2023_1852_MOESM2_ESM.pdf]

nature portfolio

Corresponding author(s): Youjun Wang

Last updated by author(s): Mar 15, 2023

Reporting Summary

Nature Portfolio wishes to improve the reproducibility of the work that we publish. This form provides structure for consistency and transparency in reporting. For further information on Nature Portfolio policies, see our Editorial Policies and the Editorial Policy Checklist.

Statistics

For all statistical analyses, confirm that the following items are present in the figure legend, table legend, main text, or Methods section.

n/a	Confirmed	
☐	☒	The exact sample size (*n*) for each experimental group/condition, given as a discrete number and unit of measurement
☐	☒	A statement on whether measurements were taken from distinct samples or whether the same sample was measured repeatedly
☐	☒	The statistical test(s) used AND whether they are one- or two-sided<br>*Only common tests should be described solely by name; describe more complex techniques in the Methods section.*
☒	☐	A description of all covariates tested
☒	☐	A description of any assumptions or corrections, such as tests of normality and adjustment for multiple comparisons
☐	☒	A full description of the statistical parameters including central tendency (e.g. means) or other basic estimates (e.g. regression coefficient) AND variation (e.g. standard deviation) or associated estimates of uncertainty (e.g. confidence intervals)
☐	☒	For null hypothesis testing, the test statistic (e.g. *F*, *t*, *r*) with confidence intervals, effect sizes, degrees of freedom and *P* value noted<br>*Give P values as exact values whenever suitable.*
☒	☐	For Bayesian analysis, information on the choice of priors and Markov chain Monte Carlo settings
☒	☐	For hierarchical and complex designs, identification of the appropriate level for tests and full reporting of outcomes
☒	☐	Estimates of effect sizes (e.g. Cohen's *d*, Pearson's *r*), indicating how they were calculated

Our web collection on statistics for biologists contains articles on many of the points above.

Software and code

Policy information about availability of computer code

Data collection	Data of confocal imaging and iPEAQ measurements were collected with Zen 2.1 software. All time-lapse fluorescence imaging were carried out using SlideBook 6.0.23. Results of biophysical characterization were collected by UVProbe, Fluoracle and SoftMax Pro v7.x. Data of Ca2+ imaging in hippocampal neurons were collected with NIS-Elements AR 5.10.00. Two-photon laser Ca2+ imaging were performed with scanbox 4.1 or FV10-ASW 4.2, and electrophysiology data were collected by pClampex 10.3. Visual stimulus was controlled by LabVIEW 8.5. Optical fiber recording was achieved with ORFS V2_14397.
Data analysis	The acquired images or data were analyzed by MATLAB 2014a or OLYMPUS FV10-ASW and plotted with Prism7 software. We mostly used customized Matlab scripts for very basic matrix operations, such as extracting data points from excel spread sheets that contain raw data, calculations, and commonly used statistical analysis. The related codes can be shared upon reasonable requests.

For manuscripts utilizing custom algorithms or software that are central to the research but not yet described in published literature, software must be made available to editors and reviewers. We strongly encourage code deposition in a community repository (e.g. GitHub). See the Nature Portfolio guidelines for submitting code & software for further information.

Data

Policy information about availability of data

All manuscripts must include a data availability statement. This statement should provide the following information, where applicable:

- Accession codes, unique identifiers, or web links for publicly available datasets
- A description of any restrictions on data availability
- For clinical datasets or third party data, please ensure that the statement adheres to our policy

All data generated or analyzed during this study are included in this published article (and its supplementary information files). Source Data are available with this publication. Key NEMO plasmids are available via Addgene (189930 ~ 189934). The coding sequence of NEMO sensors have been deposited to GenBank (NEMOf, OQ626715; NEMOc, OQ626716; NEMOb, OQ626717; NEMOm, OQ626718; NEMOs, OQ626719).

Human research participants

Policy information about studies involving human research participants and Sex and Gender in Research.

Reporting on sex and gender	N/A
Population characteristics	N/A
Recruitment	N/A
Ethics oversight	N/A

Note that full information on the approval of the study protocol must also be provided in the manuscript.

Field-specific reporting

Please select the one below that is the best fit for your research. If you are not sure, read the appropriate sections before making your selection.

☒ Life sciences ☐ Behavioural & social sciences ☐ Ecological, evolutionary & environmental sciences

For a reference copy of the document with all sections, see nature.com/documents/nr-reporting-summary-flat.pdf

Life sciences study design

All studies must disclose on these points even when the disclosure is negative.

Sample size	We determined sample size based on well established studies in the field and the animal-to-animal variability observed during the experiments. The sample size in detecting the performance of transiently expressed NEMO sensors in non-excitable HEK-293 cells was at least 3 times repeats with 9~20 cells per repeat. The sample size in detecting the responses of NEMO variants in dissociated rat hippocampus neurons excited by electric field stimulation was at least 10 neurons in three different primary hippocampal neuron cultures. The sample size of tail-pinching stimulus and optical fiber recording was 97~101 cells from 3 mice. The sample size of simultaneous two-photon laser Ca2+ imaging and electrophysiology in visual cortical slices was 40~223 cells from at least 3 mice. Corresponding literatures provided in methods.
Data exclusions	No data were excluded from the analyses.
Replication	At least three labs have independently confirmed the superior SBR of NEMO sensors. Experiments have been done with several mice or cells to check the reproducibility of our results. At least three independent repeats were carried out for each set of experiments.
Randomization	Allocation of samples was random.
Blinding	The investigators were blinded to group allocation during data collection and analysis.

Reporting for specific materials, systems and methods

We require information from authors about some types of materials, experimental systems and methods used in many studies. Here, indicate whether each material, system or method listed is relevant to your study. If you are not sure if a list item applies to your research, read the appropriate section before selecting a response.

Materials & experimental systems

n/a	Involved in the study
☒	☐ Antibodies
☐	☒ Eukaryotic cell lines
☒	☐ Palaeontology and archaeology
☐	☒ Animals and other organisms
☒	☐ Clinical data
☒	☐ Dual use research of concern

Methods

n/a	Involved in the study
☒	☐ ChIP-seq
☒	☐ Flow cytometry
☒	☐ MRI-based neuroimaging

Eukaryotic cell lines

Policy information about cell lines and Sex and Gender in Research

Cell line source(s)	Hek 293 cells used in this experiment were derived from ATCC (American Type Culture Collection, cat#: crl-1573). HeLa cells also from ATCC (cat#: CL 0101).
Authentication	Authentication was guaranteed by the provider. We verified the cell lines based on morphology.
Mycoplasma contamination	The cell lines were not tested for mycoplasma contamination.
Commonly misidentified lines (See ICLAC register)	The study did not involve commonly misidentified cell lines.

Animals and other research organisms

Policy information about studies involving animals; ARRIVE guidelines recommended for reporting animal research, and Sex and Gender in Research

Laboratory animals	E18 Wistar rats of either gender, C57BL/6 mice at postnatal 15-20 days (P15-P20) and adult mice(> P50) of either gender and male C57BL/6 mice (7 weeks old, weighing 20-25 g). C57BL/6 mice were housed in a 12 h light/dark cycle. Food and water were provided ad libitum. The temperature of the room was controlled at 20–25 °C, and the humidity was maintained at 45–60%.
Wild animals	The study did not involve wild animals.
Reporting on sex	Male or female mice/rats were used randomly
Field-collected samples	The studies did not involve samples collected in field.
Ethics oversight	Animal experiments were approved by the Animal Experimental Ethics Committee of Beijing Normal University and University of Science and Technology of China.

Note that full information on the approval of the study protocol must also be provided in the manuscript.
