# Supplementary figures and images for: Engineering of NEMO as calcium indicators with large dynamics and high sensitivity
Source: Nat Methods. 2023 Apr 20;20(6):918–24. doi: 10.1038/s41592-023-01852-9 (PMC10250196; doi:10.1038/s41592-023-01852-9)

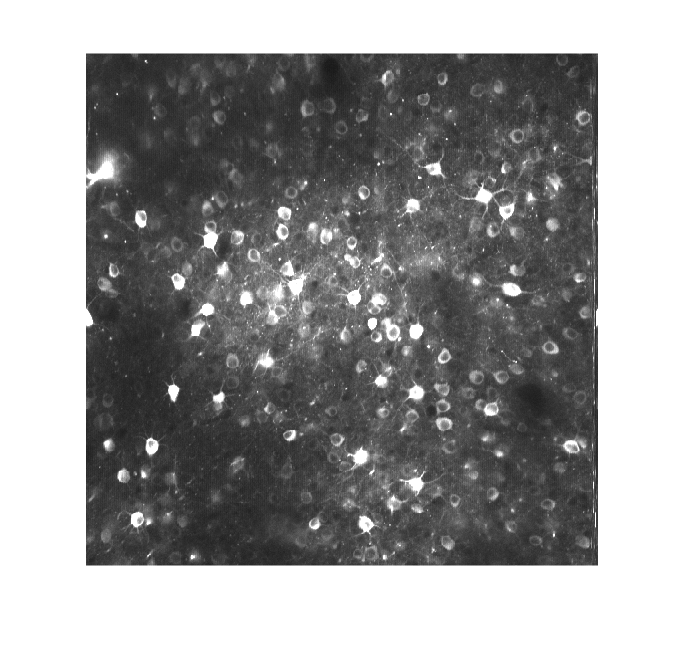

Supplement: Source Data Figs. 1–4 and Extended Data Figs. 2–9 — Source data [file 41592_2023_1852_MOESM8_ESM.zip › Source_Data_Extended_Data_Fig.9A.tif]
